# Delayed bilateral vocal cord paresis after a continuous interscalene brachial plexus block and endotracheal intubation: A lesson why we should use low concentrated local anesthetics for continuous blocks

**DOI:** 10.1097/MD.0000000000006598

**Published:** 2017-04-14

**Authors:** Hee-Sun Park, Ha-Jung Kim, Young-Jin Ro, Hong-Seuk Yang, Won-Uk Koh

**Affiliations:** Department of Anesthesiology and Pain Medicine, Asan Medical Center, University of Ulsan College of Medicine, Seoul, Korea.

**Keywords:** interscalene, peripheral nerve block, recurrent laryngeal nerve, vocal cord paresis

## Abstract

**Rationale:**

Recurrent laryngeal nerve block is an uncommon complication that can occur after an interscalene brachial plexus block (ISB), which may lead to vocal cord palsy or paresis. However, if the recurrent laryngeal nerve is blocked in patients with a preexisting contralateral vocal cord palsy following neck surgery, this may lead to devastating acute respiratory failure. Thus, ISB is contraindicated in patients with contralateral vocal cord lesion. To the best of our knowledge, there are no reports of bilateral vocal cord paresis, which occurred after a continuous ISB and endotracheal intubation in a patient with no history of vocal cord injury or surgery of the neck.

**Patient concerns:**

A 59 year old woman was planned for open acromioplasty and rotator cuff repair under general anesthesia. General anesthesia was induced following an ISB using 0.2% ropivacaine and catheter insertion for postoperative pain control.

**Diagnoses:**

While recovering in the postanesthesia care unit (PACU), however, the patient complained of a sore throat and hoarseness without respiratory insufficiency. On the morning of the first postoperative day, she still complained of mild dyspnea, dysphonia, and slight aspiration. She was subsequently diagnosed with bilateral vocal cord paresis following an endoscopic laryngoscopy examination.

**Interventions:**

The continuous ISB catheter was immediately removed and the dyspnea and hoarseness symptoms improved, although mild aspiration during drinking water was still present.

**Outcomes:**

On the 4th postoperative day, a laryngoscopy examination revealed that the right vocal cord movement had returned to normal but that the left vocal cord paresis still remained.

**Lessons:**

When ISB is planned, a detailed history-taking and examination of the airway are essential for patient safety and we recommend that any local anesthetics be carefully injected under ultrasound guidance. We also recommend the use of low concentration of local anesthetics to avoid possible paralysis of the vocal cord.

## 1 Introduction

A continuous interscalene brachial plexus block (ISB) is widely used for postoperative pain control following a shoulder operation.^[1]^ There are several known complications which can arise following ISB, although many of them are known to be transient and not lead to significant deficits.^[2–4]^ Severe life-threatening complications are also possible, however, and care must be taken to prevent such adverse events.^[5,6]^ Recurrent laryngeal nerve block is an uncommon complication that can occur after ISB, which may lead to vocal cord palsy or paresis. The majority of cases are known to be unilateral and arise on the ipsilateral side of the procedure.^[7,8]^ However, if the recurrent laryngeal nerve is blocked in patients with a preexisting contralateral vocal cord palsy following neck surgery, this may lead to devastating acute respiratory failure. Thus, ISB is contraindicated in patients with contralateral vocal cord lesion. Here, we report an exceptional case of bilateral vocal cord paresis following continuous ISB and endotracheal intubation in a patient scheduled for open rotator cuff repair surgery, who had no preexisting risk factors or history of contralateral vocal cord paresis.

## 2 Case report

A 59-year-old woman of 146 cm in height and 53 kg in weight was diagnosed with a right rotator cuff tendon tear and was scheduled for an open acromioplasty and rotator cuff repair under general anesthesia. The patient's medical history included hypothyroidism of unknown origin, rheumatoid arthritis involving her hands and no previous surgeries. Her preoperative examination and laboratory data were all within normal limits other than a slight sinus bradycardia, and her heart rate was 57 beats per minute on an electrocardiogram. The American Society of Anesthesiologists classification of her physical status was II. A preoperative airway evaluation revealed adequate mouth opening with a Mallampati score of grade II. The patient had normal dentition; adequate neck motion without limitation and her thyromental distance was more than a 3 finger width. There were no symptoms of shortness of breath, hoarseness, or any history of voice change. The anesthetic plan for this patient was general anesthesia with endotracheal intubation and mechanical ventilation under inhaled anesthetics with continuous ISB for preemptive and postoperative analgesia.

**Figure 1:**
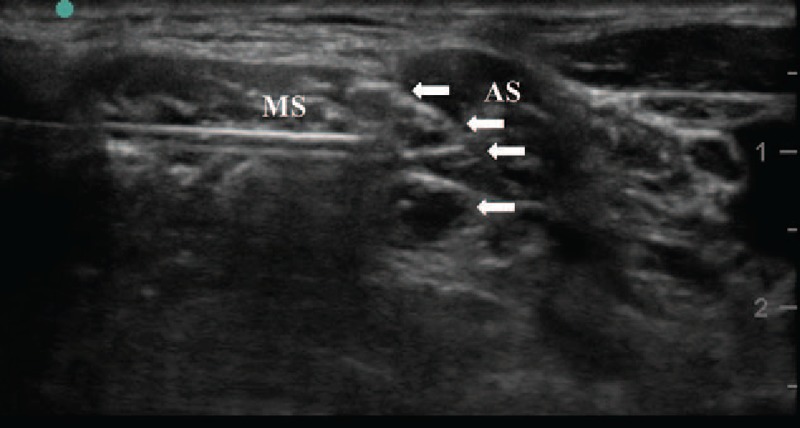
Ultrasound view of the interscalene block. The needle tip is positioned immediately lateral to the upper roots of the brachial plexus. Arrows: nerve roots of the brachial plexus, AS = anterior scalene muscle, MS = middle scalene muscle.

In the operating room, basic monitors consisted of a 3-lead electrocardiogram, and pulse oximetry, noninvasive blood pressure, and capnography were applied. Then, 1 mg of midazolam and 50 μg of fentanyl were intravenously injected for the patient comfort during ISB procedure. Before induction of general anesthesia, a right side ISB was performed with the patient in the left lateral position. Under all aseptic precautions, the brachial plexus was identified between the right anterior and middle scalene muscles using a 6 to 15 MHz linear array transducer (HGL50x; Sonosite, Bothell, WA). Two percent lidocaine was injected to anesthetize skin approximately 2 to 3 cm from the edge of the transducer. An 18-gauge 50-mm Tuohy stimulating needle (StimuLong NanoLine: Pajunk GmbH, Geisingen, Germany) was introduced in-plane under ultrasound guidance with a posterior approach^[9,10]^ through the middle scalene muscle. The successful position of the needle tip was identified immediately posterior to the interscalene brachial plexus nerve roots and was further confirmed by a nerve stimulator (Fig. 1). The stimulation current was started at 0.5 mA and, once the desired stimulation response was elicited, 10 mL of 0.2% ropivacaine was injected with intermittent aspiration under real-time ultrasound guidance. Following this injection, a continuous catheter for postoperative pain control was advanced approximately 1 to 2 cm beyond the needle tip and its appropriate position adjacent to the brachial plexus nerve roots was confirmed with a 1- to 2-mL injection of local anesthetics. The catheter was tunneled subcutaneously and then sutured using 4–0 nylon and a transparent aseptic dressing was applied. An ipsilateral skin sensory decrease at the upper shoulder and decrease in motor strength during shoulder abduction confirmed a successful block. Immediate complications were also evaluated after nerve block injection and indwelling catheter placement and the patient showed no signs or symptoms of respiratory failure such as dyspnea and hoarseness.

Following the ISB with continuous catheter placement, general anesthesia was induced with lidocaine (40 mg) and propofol (80 mg) and tracheal intubation was facilitated with rocuronium (40 mg). The vocal cord of the patient was fully exposed under direct laryngoscopy and endotracheal intubation was successfully performed without difficulty or trauma. A 7.0-mm silastic endotracheal tube was inserted and fixed at a depth of 19.5 cm from the incisor teeth after confirmation of equal bilateral lung sounds. Anesthesia was maintained with desflurane (6%–7%) with oxygen and nitrous oxide (50%), with the fresh gas flow set at 1.2 L/min. The shoulder surgery was performed in the beach chair position, and the patient's vital signs remained stable throughout the procedure. The lowest measured blood pressure and heart rate during anesthesia were 90/50 mmHg and 55 beats per minute, respectively.

After the end of surgery, a second 10-mL bolus of 0.2% ropivacaine was injected through the catheter for immediate postoperative pain management and to confirm good catheter function. A nerve-blocking patient-controlled analgesia (PCA) consisting of 0.2% ropivacaine was connected before emergence. The PCA was set at a continuous infusion of 5 mL and bolus infusion of 3 mL with a lockout time of 20 minutes and a total volume of 250 mL. Intravenous glycopyrrolate (0.4 mg) with neostigmine (2.0 mg) was administered to reverse the neuromuscular blockade. The trachea was extubated when the patient was awakened with adequate spontaneous respiration. There was no sign of any respiratory defect or difficulty immediately after extubation. The total operation time was 114 minutes and the total general anesthesia time was 158 minutes from intubation to extubation.

The patient was transferred to the postanesthesia care unit (PACU) after emergence, but subsequently complained of a sore throat and hoarseness. Horner's syndrome was not observed. Because the patient did not present with any respiratory insufficiency or shortness of breath, and the pulse oximeter showed an oxygen saturation of >96% at room air with all other vital signs within normal range, we regarded this as a minor complication of the endotracheal intubation and the patient was reassured that it might be because of a mucosal injury from the intubation. Her pain score of the surgical site was 0 on the numerical rating scale. The patient was evaluated with the modified Aldrete scoring system and was given a score of 9, which indicated a sufficient recovery to be transferred from the PACU to the general ward. Upon transfer to the general ward, the hoarseness and sore throat symptoms remained overnight and the patient also complained of mild breathing discomfort. However, there were no signs of acute respiratory obstruction or desaturation at the general ward.

**Figure 2:**
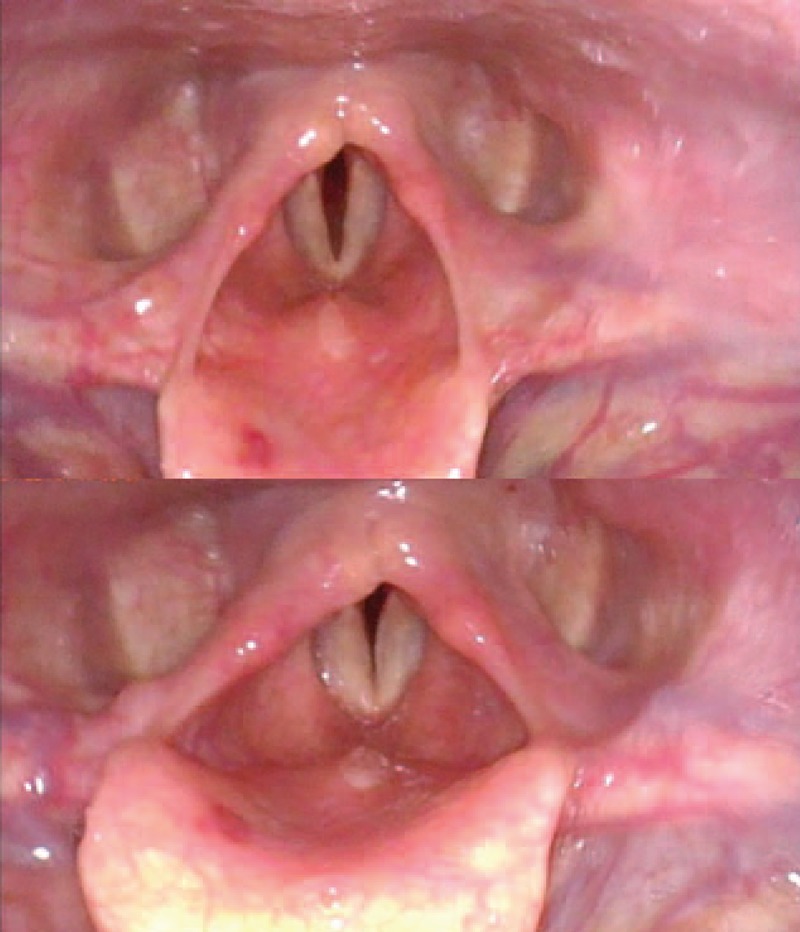
Endoscopic laryngoscopic image on the first postoperative day showed bilateral vocal cord paresis with only a 2-mm gap.

On the morning of the first postoperative day, the patient still complained of mild dyspnea, dysphonia, odynophagia, and slight aspiration when swallowing water. An otolaryngologist examined her upper airway using endoscopic laryngoscopy and diagnosed paresis of both vocal cords with only a 2-mm gap observed between them (Fig. 2). Vocal cord edema was not observed. The continuous ISB catheter was immediately removed and the dyspnea and hoarseness had improved within 2 hours, although mild aspiration when drinking water was still present.

**Figure 3:**
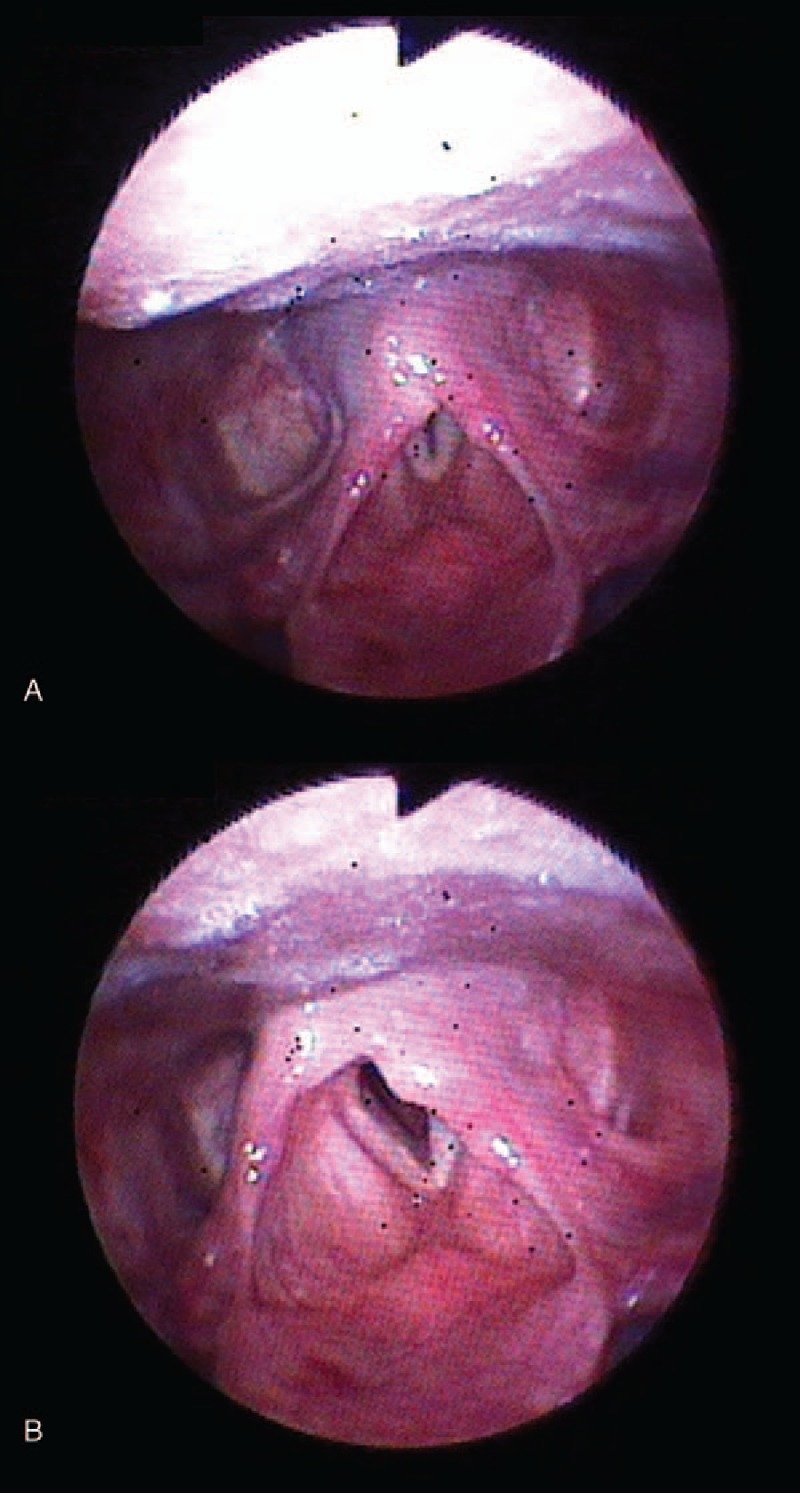
(A) Both cords meet in the midline on phonation. (B) During inspiration, there was a left median fixation indicating left vocal cord palsy on an endoscopic laryngoscopic examination on the 4th postoperative day. Because the gap was reduced, the patient's aspiration symptoms improved.

On the fourth postoperative day, the right vocal cord movement had returned to normal but the left vocal cord paresis still remained upon laryngoscopy examination. (Fig. 3) Her subjective symptoms had all improved but mild hoarseness and aspiration still persisted. She was discharged without any other complications and with an appointment for the otolaryngology outpatient clinic.

Upon visiting the otolaryngology outpatient clinic one month later, the patient still complained of mild aspiration when drinking water, hoarseness, and a foreign body sense in the throat. A laryngeal nerve electromyography (EMG) test demonstrated persistent left recurrent laryngeal neuropathy.

## 3 Discussion

We have reported a case of bilateral vocal cord paresis, which occurred following continuous ISB and general anesthesia with endotracheal intubation. Considering the symptom and course of this patient and the results of an otolaryngology examination, we assessed that the ipsilateral vocal cord paresis was caused by the continuous ISB and that trauma following or during endotracheal intubation had caused the contralateral lesion.

Continuous ISB is a useful method for postoperative pain control after shoulder surgery, and compared with intravenous PCA with opioids, the continuous ISB technique is known to provide better pain control, a lower incidence of side effects, and a higher degree of patient satisfaction with a significant opioid sparing effect.^[11,12]^ However, caution is needed during ISB as several complications can arise.^[2]^ Recurrent laryngeal nerve palsy is a known complication of ISB, with an incidence of 3% to 6%. When the recurrent laryngeal nerve is blocked following ISB, it is known to normally present symptoms within 30 minutes of the initial bolus.^[6]^ Complications of an ipsilateral recurrent laryngeal nerve block usually have less clinical implications, such as mild hoarseness. However, there are reported cases which presented with acute respiratory failure owing to bilateral vocal cord paresis after ISB.^[5,6,13]^ Plit et al^[5]^ described a case of bilateral vocal cord paresis following ISB in a patient who had previously undergone a total thyroidectomy and had symptoms of persistent mild hoarseness. Solanski et al^[6]^ also reported a case of acute respiratory difficulty after a right-side supraclavicular brachial plexus block in a patient with an unrecognized preexisting left side vocal cord palsy. To our knowledge, however, no previous report has documented bilateral vocal cord paresis resulting from ISB and endotracheal intubation in a patient with no preexisting vocal cord palsy or previous neck-associated surgical history, which we report in our current case.

The close proximity of neurovascular structures to the areas affected by ISB contributes to most of the reported side effects and complications.^[7]^ The right and left recurrent laryngeal nerves arise from the vagus nerve. The right recurrent laryngeal nerve hooks backwards and upward behind the subclavian artery and ascends to the trachea-esophageal groove. The left recurrent laryngeal nerve crosses the arch of the aorta in the thorax, hooks beneath the arch, and ascends into the neck in the left trachea-esophageal groove.^[5]^ During their ascent from the thorax to the larynx, numerous anatomic variations may be encountered.^[14]^ The anatomic position and variations of the recurrent laryngeal nerves and the anterior spread of the local anesthetics over the anterior scalene muscle are responsible for the recurrent laryngeal nerve involvement after ISB.^[5]^ When a continuous peripheral nerve block catheter is placed too anteriorly within the interscalene groove or if the catheter migrates in a cephalad manner, the local anesthetics delivered through the catheter may affect the area of the recurrent laryngeal nerve pathway. A recurrent laryngeal nerve block after ISB is usually self-limiting, and indeed the right-sided, ipsilateral vocal cord paresis of our current case was relieved promptly after discontinuation of the right side continuous ISB.

Another possible explanation for the recurrent laryngeal nerve block in our current patient may have been a prevertebral fascial defect. Winnie and Winnie et al^[15,16]^ write that the fascia covering the scalene muscles is derived from the prevertebral fascia, the posterior fascia of the anterior scalene muscle, and the anterior fascia of the middle scalene muscle that constitute the sheath of the brachial plexus.^[17]^ If this prevertebral sheath has a defect, local anesthetics could spread toward the anterior area and subsequently paralyze the recurrent laryngeal nerve thereby leading to symptoms of paresis or palsy of the vocal cord. Moreover, if a certain amount of anterior spread of local anesthetics occurs after ISB or a supraclavicular brachial plexus block, Horner syndrome could arise together with vocal cord paresis, considering the anatomical position of the cervical sympathetic ganglion. However, we did not observe this syndrome in our present patient, possibly because of the low concentration of local anesthetics that was used (0.2% ropivacaine).

During the preoperative examination, our current patient was found to have no surgical history at all and no symptoms of vocal cord paresis/palsy. However, bilateral vocal cord paresis occurred after continuous ISB combined with general anesthesia. After removal of the continuous ISB catheter, the right side vocal cord movement was recovered, but the left side (contralateral side of the ISB) vocal cord paresis persisted. This may be because the bilateral vocal cord paresis had occurred secondary to the right recurrent nerve block from the right-sided continuous ISB combined with left recurrent laryngeal nerve injury from a different pathology other than ISB. These pathologies could have been idiopathic^[13]^ or an unknown preexisting vocal cord lesion or trauma resulting from the endotracheal tube insertion. Our patient had no previous surgical history or intervention to the neck or airway including an endotracheal intubation. Furthermore, laryngoscopic examination during the induction of anesthesia did not reveal any significant lesions in the vocal cords and a postoperative laryngeal examination did not demonstrate any lesion or pathologic condition other than recurrent laryngeal nerve palsy.

Considering the findings and clinical course of our patient, we strongly consider that the left side recurrent laryngeal nerve paresis in this case was because of traumatic injury of the vocal cord following endotracheal intubation. Unilateral vocal cord palsy may possibly result from asymmetrical inflation of the endotracheal tube cuff at the level of the subglottic larynx,^[18,19]^ or other causes of nerve damage by the endotracheal tube may have occurred because of an unsuitable position during surgery or compression of the airway because of movement of this position. Our patient was placed in the beach chair position during surgery, and there has been possible mechanical injury to the vocal cord during this positioning such as endotracheal tube traction. Additionally, endotracheal tube cuff pressure may have been increased by diffusion of nitrous oxide into the cuff.^[19]^

Our present patient did not show acute respiratory failure other than hoarseness in the immediate postoperative period in the PACU. There is a previous case report of delayed vocal fold paralysis after continuous ISB with catheter placement that occurred approximately 8 hours after the initial local anesthetics bolus through the ISB catheter.^[7]^ Although our current case differed from that previous patient, she did show delayed aggravation and prolonged bilateral vocal cord paresis signs and symptoms. We speculated that the mild initial symptoms in the PACU were because of the left recurrent laryngeal nerve paresis following trauma from the endotracheal intubation and the delayed aggravation was because of the right recurrent laryngeal nerve paresis following accumulation of local anesthetic through the continuous ISB PCA.

Nigel et al^[20]^ have also reported that the presence of unrecognized contralateral recurrent laryngeal nerve palsy owing to cancer in a female patient had a postextubation stridor after an interscalene block with 30 mL of 0.375% bupivacaine combined with general anesthesia. The oxygen saturation in that previous patient decreased from 99% to 82%, and required reintubation and eventually a tracheostomy. However, our current patient did not show acute respiratory failure or desaturation, possibly because the concentration and volume of local anesthetics we used were lower than that in the patient described by Nigel et al and other previous reports. Furthermore, accumulation of the relatively low-concentrated local anesthetics (0.2% ropivacaine) used in our continuous ISB PCA regimen may have contributed to this delayed onset. Thus, when ISB is used as an adjunct to general anesthesia for shoulder surgery and continuous ISB is further planned for postoperative analgesia, it is not necessary to use high-concentrated local anesthetics which may induce complete motor block. Previous studies have suggested that ISB with low-concentration local anesthetics (0.25% or 0.2% ropivcaine) can effectively achieve successful postoperative analgesia with minimal impairment to hand grip motor strength and pulmonary function.^[21,22]^ Furthermore, the magnitude of systemic toxicity may be minimized.

## 4 Conclusions

When ISB is planned, a careful history-taking and examination of the airway are essential for the safety of the patient and we recommend careful injection of the local anesthetics under ultrasound guidance to minimize their anterior spread during the procedure. Furthermore, if general anesthesia is to be combined with ISB or continuous ISB catheterization is planned, we highly recommend the use of low-concentrated local anesthetics to avoid possible complete paralysis of the vocal cord.
